# European Public Health News

**DOI:** 10.1093/eurpub/ckad074

**Published:** 2023-06-01

**Authors:** Marieke Verschuuren, Hans Henri P Kluge, Nino Berdzuli, Francesca Racioppi, Anthony Staines, Floris Barnhoorn

**Affiliations:** EUPHA; WHO Regional Office for Europe; WHO Regional Office for Europe; WHO European Centre for Environment and Health; (EPH Conference); WHO Regional Office for Europe

In this edition of the European Public Health News, the EUPHA office column puts the spotlight on EUPHA’s sections. These thematic networks are an important element of the EUPHA organization. The contribution of WHO Regional Office for Europe focuses on environmental determinants. The health sector has a vital role to play in what lies ahead. It can address environmental challenges and must proactively lead in the search for solutions. Finally, Staines and Barnhoorn of the European Public Health Conference invite you to the 2023 conference in Dublin and provide a first glimpse of the plenary programme.

## Tackling environmental determinants must be at the fore of the public health agenda

KlugeHans Henri P
WHO Regional Office for Europe
BerdzuliNino
WHO Regional Office for Europe
RacioppiFrancesca
WHO European Centre for Environment and Health

As the European Region jumps from one health emergency to the next in what has been coined a ‘permacrisis’, national health systems are struggling to deal with multiple, often overlapping emergencies—while also providing quality health care.

Today’s public health agenda is a challenging one, driven mainly by the rapid ageing of the region’s population and the heavy burden of non-communicable diseases, of which environmental risks are significant determinants.

One of these determinants is air pollution; responsible for an estimated 724 000 premature deaths per year in the WHO European Region, based on 2019 data, contributing to almost 30% of mortality from lung cancer, 24% from strokes, 25% from cardiovascular diseases and more than 40% of mortality from lung disease.

Crises bring about change. Nothing has exposed our collective systemic failures to prevent and address environmental factors that increase the risk of zoonotic diseases, as the COVID-19 pandemic has. It has highlighted how much health and well-being are intrinsically linked to and affected by the triple environmental crisis caused by climate change, pollution, land degradation and biodiversity loss. These three interlinked issues reinforce each other and drive further damage to the health of humans and the planet alike.

With the hottest summer on record just behind us, a trail of deaths from heatwaves and wildfires, and new evidence that Europe is warming at twice the global average speed, the climate crisis is now our reality. It is quite simply the greatest threat of our time, causing death and illness from more frequent extreme weather events, the disruption of food systems, increases in zoonoses and food, water and vector-borne diseases, as well as mental health issues.

A leading driver of zoonotic diseases that may cause pandemics, is loss of biodiversity. As we lose biodiversity and ecosystems deteriorate as a result of pollution, deforestation, land degradation and desertification, this in turn, directly impacts human health. Environmental pollution caused by energy production, industrial processes, transportation and agricultural practices can lead to a wide range of diseases. Preserving nature, on the other hand, protects human health. What is more; sustainably balancing and optimizing the health of animals and ecosystems, also optimizes human health.

The complexities we now face, associated with simultaneous, intertwined challenges—can only be dealt with through an integrated, cross-sectoral approach, a central component of the European Programme of Work 2022–2025 ‘United Action for Better Health for Europe’, serving as the main driver for sustainable development and ‘building forward better’. The Programme of Work incorporates approaches such as One Health, recognizing that the health of humans, animals, plants and the environment are one and the same; intricately linked.

The health sector has a vital role to play in what lies ahead. It can address environmental challenges and must proactively lead in the search for solutions. Improving the environments people inhabit, decreases the disease burden, reduces inequalities, relieves pressured health systems while increasing their resilience, and accelerates the attainment of the Sustainable Development Goals. Preparing health systems to respond effectively to environmental emergencies and ensuring their resilience to climate change, saves lives and cuts costs. Health systems that protect the environment and biodiversity are a part of the solution.

A Ministerial Conference on Environment and Health, in Budapest, Hungary, on 5–7 July 2023, will provide a unique opportunity to reshape countries’ actions and give the environment the prominence it deserves in health systems and across government policies to ensure thriving, safe and health-nurturing environments for the Region’s nearly one billion people. It will address the gap between policy and action, offering a roadmap on how to work in a transformative way to implement coherent, evidence-informed policies that address health, the environment and all their determinants.

The Conference proposes a set of commitments to achieve a new public health agenda and will launch a new mechanism to promote partnerships to accelerate action on priorities of common interest. By working together, the health and environment sectors can deliver more integrated, effective policies, including improved governance for intersectoral collaboration. The legacy of our decisions will be instrumental in securing the future of the whole European Region—stretching across 53 countries comprising Europe and Central Asia—with a lasting impact for generations to come.

When we all pull in the same direction, there are no limits to what we can achieve. The Budapest Ministerial Conference will be a turning point in accelerating our actions to ensure healthier people, a thriving planet and a sustainable future.

## Our Food, Our Health, Our Earth: A Sustainable Future for Humanity

StainesAnthony
(EPH Conference)
BarnhoornFloris
WHO Regional Office for Europe

16th European Public Health Conference

8–11 November 2023

Convention Centre Dublin (The CCD), Dublin, Ireland

Over the next decade, choices will be made which will determine the future of our advanced technological civilization. We now have no way out in terms of climate emergency. The global climate is rapidly deteriorating. We know what to do, to deliver a future for our society, but it’s less clear that we will do it. COP27 achieved little in the ambition to keep the 1.5° limit alive and pull humanity back from the climate cliff. Building a future, any kind of future, for us and our children, demands a new attention to sustainability.

The EPH Conference in Dublin hopes to open part of this discussion, with a focus on sustainability, food and health. We will look specifically at global health, the use of digital tools, equity in health and the necessary staffing and skills to provide good care. We will, in line with the overall conference theme, look in depth at the climate emergency, and very specifically at the human food supply.

We look forward to seeing you all in Dublin. The city is well known as a literary city, but its long history of public health in Ireland is less well known. These intersect strikingly in one man -William Wilde, society doctor, hospital founder and father of Oscar, who needs no introduction, and Willy, who was an alcoholic and a journalist. He wrote extensively on eye surgery, Irish antiquities, Irish folklore, and on the health of the Irish population. You will have a chance to discover his city, in all its brightness and darkness, as well as learning something more about making our futures.

### Plenary programme

The plenary programme is organized in collaboration with our partners and the tentative themes of the sessions are as follows.

- Transitioning to more sustainable food systems that support health and wellbeing, organized by EuroHealthNet, Thursday 9 November, 14:00–15:00 (all times mentioned are Dublin times).
- Non-communicable diseases: risk factors and commercial determinants (title tbc), organized by EUPHA and European Observatory on Health Systems and Policies, Thursday 9 November, 17:50–18:50.
- Building the future One Health workforce, organized by ASPHER and EHMA, Friday 10 November, 10:30–11:30.
- On the path to an equitable and sustainable digital future for European health systems, organized by WHO Regional Office for Europe, Friday 10 November, 14:00–15:00.
- Global health (title tbc), organized by European Commission, Saturday 11 November, 10:30–11:30.

Names of speakers and moderators will be announced shortly on our website. Read more about the plenary programme here.

We look forward to seeing you all in person, in Dublin.

